# A new species of *Bredia* (Sonerileae, Melastomataceae) from Sichuan, China

**DOI:** 10.3897/phytokeys.152.53512

**Published:** 2020-07-03

**Authors:** Jin-Hong Dai, Qiu-Jie Zhou, Ren-Chao Zhou, Ying Liu

**Affiliations:** 1 State Key Laboratory of Biocontrol and Guangdong Key Laboratory of Plant Resources, School of Life Sciences, Sun Yat-sen University, No. 135, Xin-Gang-Xi Road, Guangzhou 510275, China Sun Yat-sen University Guangzhou China

**Keywords:** *
Bredia
*, Melastomataceae, phylogeny, taxonomy

## Abstract

*Bredia hispida* (Sonerileae, Melastomataceae), a species occurring in southeastern Sichuan, China, is newly described based on morphological and molecular data. The generic placement of *B. hispida* is well supported by phylogenetic analysis and morphological characters, including basally cordate, hairy leaf blade, cymose inflorescence, basally gibbous anthers and enlarged ovary crown enclosing an inverted frustum-shaped depression. Both molecular and morphological divergence showed that *B. hispida* is well separated from its close relatives, justifying its recognition as a distinct species. The new species resembles *B. repens*, *B. changii* and *B. guidongensis* in the prostrate habit and isomorphic stamens but differs markedly in the unequal opposed leaves, the 2–4 mm long, stout bristles on the adaxial surface of leaf blade and acuminate leaf apex. *Bredia hispida* co-occurs with *B. esquirolii* in the wild. No morphologically putative hybrids between them were observed despite their overlap in flowering season. The isolating mechanism remains unclear, pending further investigation.

## Introduction

*Bredia* Blume was originally established based on *B. hirsuta* Blume, a species endemic to Taiwan and the Ryukyu islands (Blume 1849). Circumscription of this genus had long been controversial. The dispute mainly concerned whether to include *Tashiroea* Matsum. ex T. Itô & Matsum. and certain species of *Phyllagathis* Blume in *Bredia* (Diels 1924, 1932; Merrill and Chun 1940; Li 1944; Chen 1979, 1984; Hansen 1992; Chen and Renner 2007). By combining molecular phylogenetic and morphological data, recent studies have provided strong evidence for a new generic limit of *Bredia* (Zhou et al. 2019a, 2019b, 2019c). *Bredia* was redefined as excluding *Tashiroea* while incorporating seven species previously treated in *Phyllagathis* (Zhou et al. 2019b). Together with two recently published species, *Bredia* as currently circumscribed includes 23 species distributed from central and southern mainland China, Taiwan, to the Ryukyu islands and northern Vietnam (Zhou et al. 2019b; Wen et al. 2019; He et al. in press). Species of *Bredia* are characterized by the leaf blade papery, usually hairy, inflorescences cymose, umbellate, or a cymose panicle, anther basally gibbose or tuberculate, and ovary crown persistent and enlarged enclosing an inverted frustum-shaped depression.

**Figure 1.**
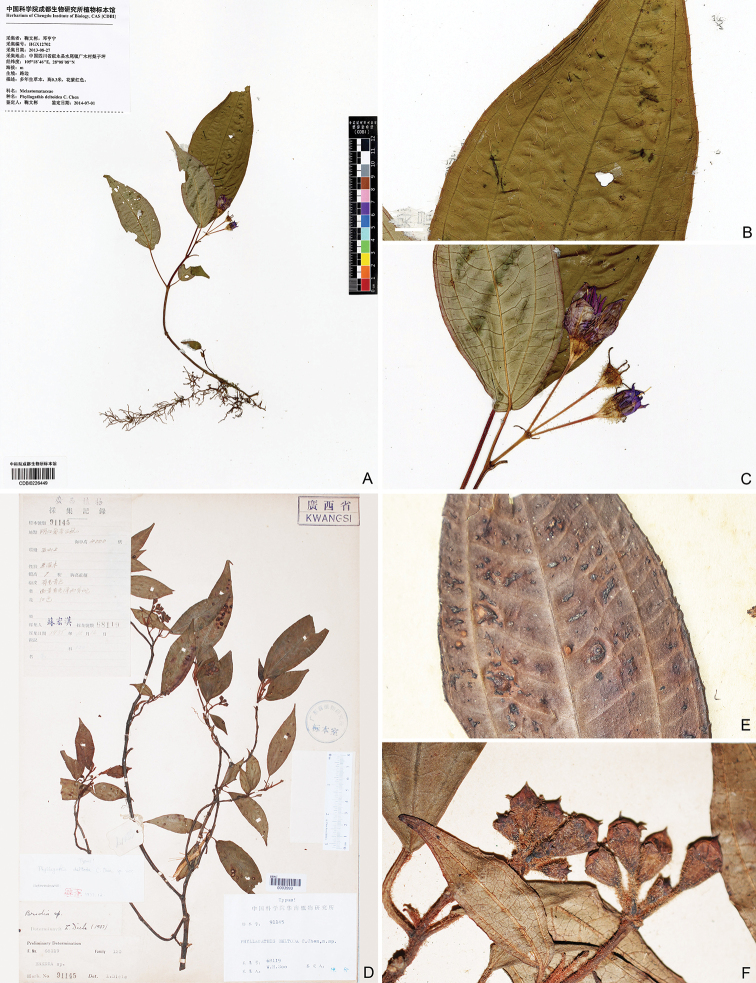
Herbarium specimen of *Bredia hispida* (**A–C**) and *Phyllagathis deltoidea* (**D–F**). **A–C** W. B. Ju and H. N. Deng, HGX12702 (CDBI) collected from Shui-wei town, Xuyong County, Sichuan, China, showing the stout long bristles on adaxial surface of the leaf blade and linear-lanceolate calyx lobes, images from National Plant Specimen Resource Center **D–F** H. H. Su 68119 (IBK, IBSC) collected from Aidian, Ningming County, Guangxi, China, holotype (IBSC) (**D, F**) and isotype (IBK) (**E**) of *Phyllagathis deltoidea*.

During a survey of specimens in Chinese herbaria for a project on species delimitation of *Bredia*, several collections from Xuyong County, southeastern Sichuan Province, caught our attention. These collections (e.g. Fig. 1A–C) were identified as *Phyllagathis deltoidea* C. Chen (Fig. 1D–F). Upon closer examination, however, the plants from Xuyong are morphologically quite different from *P. deltoidea* in having basally cordate (vs. cuneate) lamina adaxially hispid with stout long bristles (vs. puberulous and sparsely setose) and linear-lanceolate calyx lobes (vs. broadly triangular lobes) (Fig. 1). In addition, *P. deltoidea* is only recorded from its type locality in Ningming County, southwestern Guangxi, which is about 800 kilometers away from Xuyong County, southeastern Sichuan. Both morphology and distribution suggest that the current identification is erroneous.

**Figure 2.**
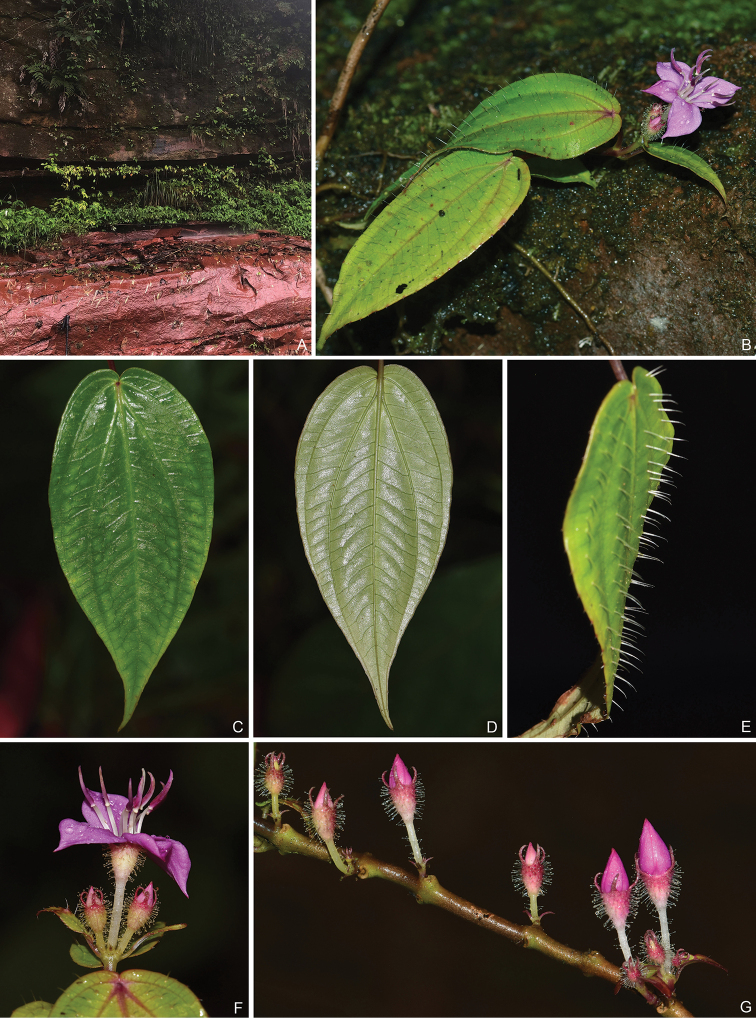
*Bredia hispida*. **A** Habitat **B** a flowering branch, showing the prostrate habit **C** adaxial leaf surface **D** abaxial leaf surface **E** lateral view of leaf, showing the stout long bristles **F** terminal inflorescence **G** axillary inflorescences on old branchlets. All from Y. Liu 764 (A, PE, SYS).

**Figure 3.**
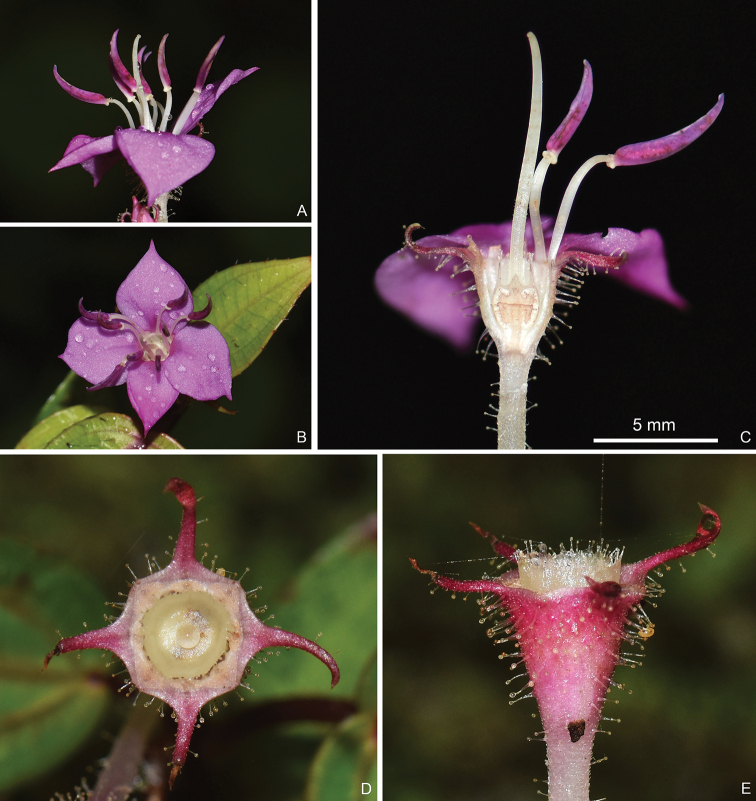
Floral details and young fruit of *Bredia hispida*. **A** Side view of a flower **B** top view of a flower **C** longitudinal section of a flower showing isomorphic stamens and ovary crown **D** top view of a young fruit **E** side view of a young fruit showing enlarged ovary crown exserted from hypanthium. Scale bar: 5 mm (**C**). All from Y. Liu 764 (A, PE, SYS).

**Figure 4.**
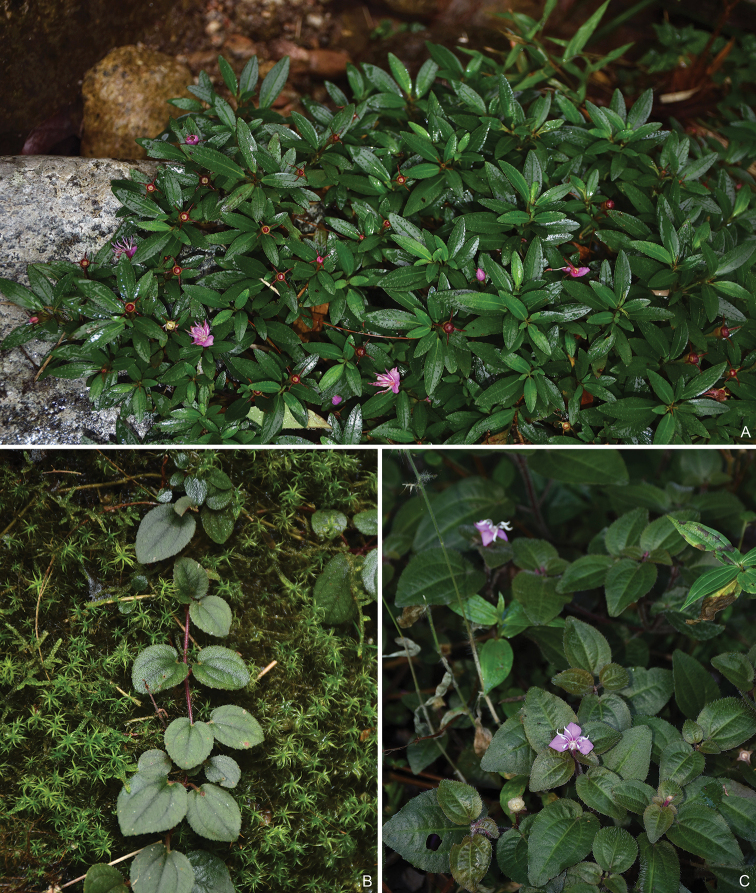
Three prostrate species of *Bredia*. **A***B. guidongensis* from Y. Liu 472 (SYS) **B***B. changii* from Y. Liu 548 (SYS) **C***B. repens* Y. Liu 558 (A, PE, SYS).

In September 2019, we made a field expedition to Xuyong County and collected flowering and fruiting specimens of the plant in question (Figs 2, 3). This plant possesses all the synapomorphies of *Bredia* aforementioned. It most closely resembles *B. changii* W. Y. Zhao, X. H. Zhan & W. B. Liao, *B. guidongensis* (K. M. Liu & J. Tian) R. Zhou & Ying Liu and *B. repens* R. Zhou, Q. J. Zhou & Ying Liu in habit and isomorphic stamens (Fig. 4), but differs markedly from the latter species in leaf morphology (Fig. 2). Judging from morphological aspects, this plant may represent an undescribed species in *Bredia*.

To test the generic affiliation of the unknown plant and its closest relative in the genus, we performed phylogenetic analyses based on DNA sequence data of nuclear ribosomal internal transcribed spacer (nrITS), sampling all species so far recorded in *Bredia*. We also calculated pairwise genetic distances among this plant and 23 species of *Bredia* to evaluate its distinctness. The results confirmed our suspicion that this plant represented a species new to science, which we described as *B. hispida* below.

## Materials and methods

To test the generic affiliation of *B. hispida* and its position in the genus, we selected ingroup taxa based on previous studies (Zhou et al. 2019a, 2019b, 2019c). The final nrITS dataset contained 35 accessions representing *Blastus* Lour., *Dissochaeta* Blume, *Fordiophyton* Stapf, *Tashiroea*, *Phyllagathis*, *Scorpiothyrsus* H. L. Li, *Blakea* P. Browne, and 23 species so far recorded in *Bredia*, with *Blakea* (Blakeeae) chosen as an outgroup according to Goldenberg et al. (2012). The sequences of *B. hispida*, *B. violacea* H. L. Li and *B. reniformis* C. M. He, Y. H. Tong & S. J. Zeng were newly sequenced, while the rest were downloaded from GenBank. The source of the materials and GenBank accession numbers are given in Supplementary material 1.

Total DNA was extracted from fresh leaves using the modified CTAB procedure (Doyle and Doyle 1987). The nrITS region of *B. hispida*, *B. violacea* and *B. reniformis* were amplified and sequenced using universal primers ITS4 and ITS5 (White et al. 1990), following the procedure described in Zou et al. (2017).

Sequences were aligned using SeqMan v.7.1.0 (DNASTAR Inc., Madison, WI). The Akaike information criterion in Modeltest version 3.7 (Posada and Crandall 1998) was used to select the best-fitting nucleotide substitution model (GTR+G) prior to phylogenetic analyses. Bayesian inference (BI), maximum likelihood (ML) and maximum parsimony (MP) analyses were performed using MrBayes 3.2.6 (Huelsenbeck and Ronquist 2001), RAxML version 8.2.10 (Stamatakis 2014) and PAUP version 4a165 (Swofford 2002) respectively. For BI analysis, two independent Markov chain Monte Carlo analyses (MCMC) were performed with four simultaneous chains of 2,000,000 generations sampling one tree every 100 generations. The first 25% of trees were discarded as burn-in and the remaining were used to construct a majority-rule consensus tree with Bayesian posterior probabilities (PP). We verified that the average deviation of split frequencies had reached a value below 0.01 at the end of MCMC analyses. We also assessed the effective sample sizes (ESS) for all parameters and statistics using Tracer version 1.7.1 (Rambaut et al. 2018). ML analyses were performed under GTR+G model as recommended by the author. Node support was estimated with 1,000 bootstrap replicates using a fast bootstrapping algorithm (Stamatakis et al. 2008). For MP analyses, a heuristic search strategy was conducted of 1000 random addition replicates, with the tree-bisection-reconnection (TBR) branch swapping algorithm and MultTrees on. Maxtree was set to 500. Node support was evaluated by 1000 bootstrap replicates of 1000 random additions. Pairwise genetic distances among *B. hispida* and species of *Bredia* were calculated using the Kimura 2-parameter method (Kimura 1980).

## Results

**Figure 5.**
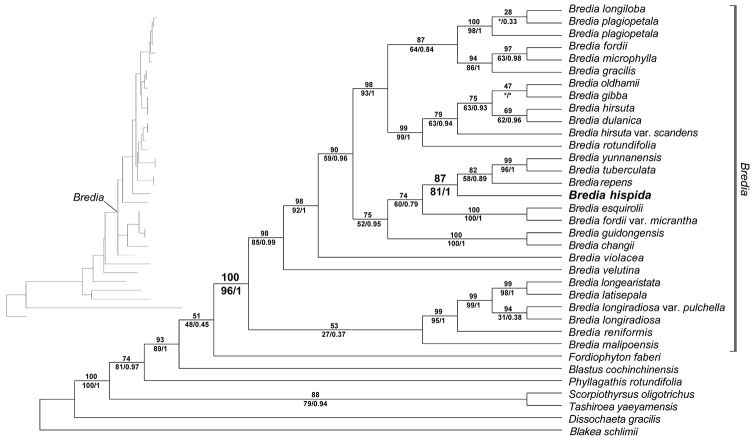
Phylogenetic position of *Bredia hispida*. Maximum likelihood (ML) phylogenetic tree based on nrITS sequence data. Numbers above branches are bootstrap values obtained from maximum likelihood analyses, and those below branches are Bayesian posterior probabilities (right) and bootstrap values (left) resulting from maximum parsimony analyses. The new species is noted in bold. Asterisk denotes a branch collapsed in Bayesian inference or maximum parsimony analyses.

The aligned sequence matrix contained 665 characters. Statistics of sequences sampled were summarized in Supplementary material 2. The tree resulting from ML analysis is shown in Fig. 5, with PP, ML bootstrap support values (BSML), and MP bootstrap support values (BSMP) labeled at nodes. *Bredia hispida* was nested within the well supported *Bredia* clade (PP = 1.0, BSML = 100%, BSMP = 96%), forming a subclade with *B. repens*, *B. tuberculata* (Guillaumin) Diels and *B. yunnanensis* (H. Lév.) Diels (PP = 1.0, BSML = 87%, BSMP = 81%). Pairwise genetic distances among *B. hispida* and 23 species of *Bredia* are provided in Supplementary material 3.

## Discussion

### Phylogenetic position and specific status of *B. hispida*

The placement of *B. hispida* in *Bredia* is supported by phylogenetic and morphological data. Our phylogenetic analyses with complete taxon sampling of *Bredia* confirmed that *B. hispida* is a member of this clade. Morphologically, its basally cordate, hairy leaf blade, cymose inflorescence, two whorls of eight isomorphic stamens, basally slightly gibbous anthers, decurrent connectives, and enlarged ovary crown during the fruiting stage all fit well within *Bredia*.

*Bredia hispida* is phylogenetically most closely related to *B. repens*, *B. tuberculata* and *B. yunnanensis*. It is a dwarf subshrub up to 15 cm tall with its middle and lower stem prostrate, which makes it easily distinguished from most species of *Bredia*, including *B. tuberculata* and *B. yunnanensis*. It closely resembles *B. guidongensis* (Fig. 4A), *B. changii* (Fig. 4B) and *B. repens* (Fig. 4C) in the prostrate habit and isomorphic stamens, but differs in leaf morphology: opposed leaves unequal, leaf blade larger (1.5–9.9 × 0.8–4 cm), stiffly papery, ovate to ovate elliptic and apically acuminate (Fig. 2B–E). Moreover, *B. hispida* is unique in the genus in its leaf blade adaxially hispid with 2–4 mm long, stout bristles (Fig. 2E), a character previously never recorded in *Bredia*. Pairwise genetic distances between *B. hispida* and remaining species of the genus range from 0.011 to 0.066, which are comparable to the distances of most species pairs in *Bredia* (0.005–0.077). *Bredia hispida* is therefore well diverged from other species of *Bredia* from a molecular perspective. Both molecular and morphological evidence justify the recognition of *B. hispida* as a distinct species.

### Co-occurrence of *B. hispida* and *B. esquirolii*

*Bredia hispida* is currently only known from Xuyong County, Sichuan Province. It co-occurs with *B. esquirolii* (H. Lév.) Lauener, a species widely distributed in Guizhou, Chongqing and Sichuan. *Bredia hispida* grows on shady red sandstone cliff of seasonal waterfall whereas *B. esquirolii* is found in bushes, under forests and also on shady cliff (but a little further away from the dripping water). Several cases of sympatry have been observed elsewhere in the genus, viz. *B. dulanica* C. L. Yeh, S. W. Chung & T. C. Hsu and *B. oldhamii* Hook. f. in Taiwan, *B. repens* and *B. latisepala* (C. Chen) R. Zhou & Ying Liu in Hunan and *B. esquirolii* and *B. tuberculata* in Sichuan. In the first two cases, the co-occurring species have non-overlapping flowering seasons and thus interspecific reproductive isolation is easily maintained; in the third case, the flowering periods overlapped, and some putative hybrid individuals were found (unpublished data). During our visit in September 2019, both *B. hispida* and *B. esquirolii* were flowering. But no morphologically putative hybrids were observed. Pre-zygotic isolation via different pollinators is not a plausible explanation as flowers of the two species are of similar size (ca. 2 cm in diameter) and both can be visited by medium to small size bees. According to previous analyses (Zhou et al. 2019c), the crown age of the branch comprising close relatives of *B. hispida*, viz. *B. esquirolii*, *B. repens*, *B. tuberculata* and *B. yunnanensis*, was only 0.66–2.61 Mya. We suspect that other intrinsic postzygotic barriers may not have enough time to fully develop among such recently diverged species. The isolation mechanism between the sympatric *B. hispida* and *B. esquirolii* remains unclear, pending further study.

## Taxonomic treatment

### Bredia hispida

Taxon classificationPlantaeMyrtalesMelastomataceae

J.H. Dai & Ying Liu sp. nov.

8F6B35DC-5251-5CB3-AF46-C283A54BE437

urn:lsid:ipni.org:names:77209989-1

Figures 2 , 3 , 6

#### Type.

China. Sichuan: Xuyong County, Shui-wei town, Guang-mu village, 1338 m, on steep rock cliff of a small waterfall, 1 Sept 2019, Ying Liu 764 (holotype: PE; isotype: A, SYS).

#### Diagnosis.

Resembles *B. changii*, *B. repens* and *B. guidongensis* in the prostrate habit and isomorphic stamens but differs from these species in its unequal leaves (vs. equal), stiffly papery leaf blade (vs. papery) hispid with 2–4 mm long, spreading stout bristles (vs. pubescent or villous with trichomes ≤ 1 mm) and acuminate apex (vs. obtuse or acute).

#### Description.

Subshrubs, up to 15 cm tall. Stems cylindrical, inconspicuously pubescent with very short, uniseriate appressed trichomes, prostrate at middle and lower parts, branched, with adventitious roots. Opposed leaves often unequal; petiole 0.6–5 cm long, inconspicuously pubescent; leaf blade ovate to ovate elliptic, larger blades 4–9.9 × 1.6–4 cm, smaller blades 1.1–5× 0.7–2.5 cm, stiffly papery, abaxial surface pale green, inconspicuously pubescent, adaxial surface green to yellowish green, inconspicuously pubescent, hispid with spreading stout white bristles (2–4 mm long) between veins, lateral veins 2 or 3 pairs, base cordate, margin inconspicuously serrulate, apex acuminate. Inflorescences terminal or axillary, sometimes on old branchlets; 2–3-flowered cyme or solitary. Peduncle 2–10 cm long, pubescent with uniseriate appressed trichomes. Flowers bisexual, radial but androecium slightly bilateral, 4-merous, rarely 5-merous. Pedicels and calyces pubescent with uniseriate appressed trichomes and multiseriate spreading glandular trichomes. Pedicels 7–16 mm long. Hypanthium cup-shaped, ca. 4 × 4 mm, pubescent with spreading glandular trichomes. Calyx lobes 4, linear-lanceolate, ca. 3–4 mm long. Petals 4, purplish pink, ovate, 7 × 5 mm, slightly oblique, apex acute. Stamens 8, isomorphic, subequal in length, 8–10 mm long. Anthers purplish, bilocular, lanceolate, ca. 4–5.5 mm long, base slightly gibbous, connective decurrent, forming a tuberculate appendage dorsally. Ovary half inferior, locules 4, placentation axillary, ovary apex with a membranous crown, crown margin ciliate with glandular trichomes. Style ca. 11 mm long, puberulous in the lower part. Young fruit cup-shaped, apex crowned, crown exserted from hypanthium. Seeds numerous, premature.

**Figure 6.**
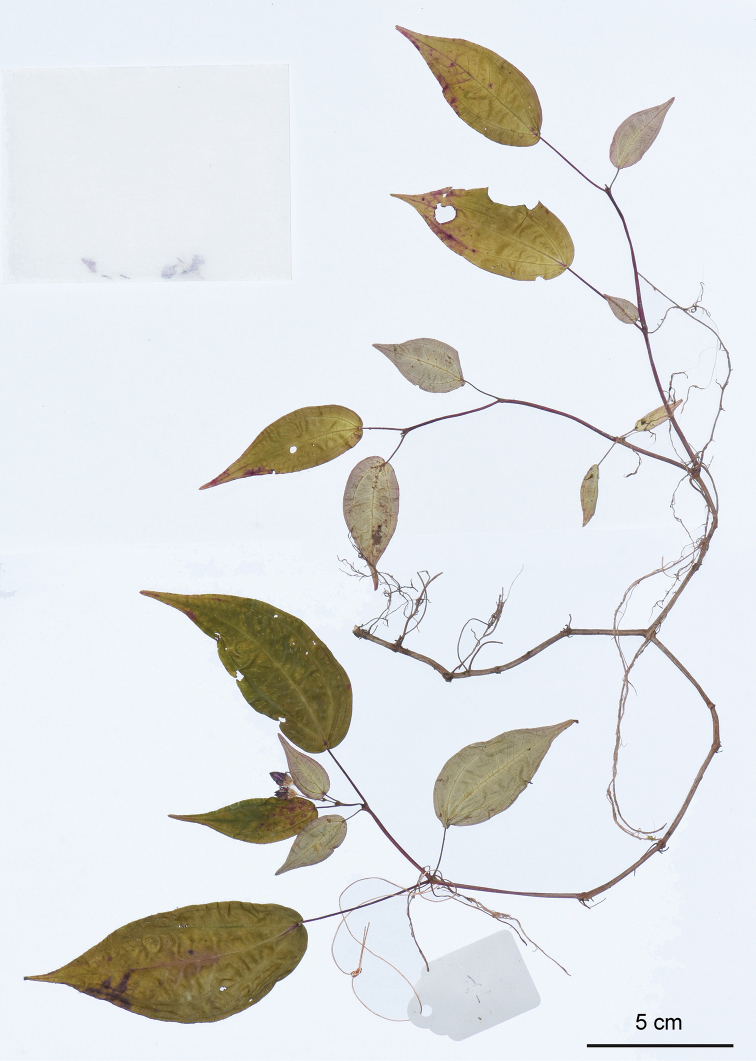
Holotype of *Bredia hispida*, Y. Liu 764 (PE). Scale bar: 5 cm.

#### Phenology.

Flowering July–September, young fruits in September.

#### Etymology.

The specific epithet is based on the spreading stout bristles on the leaf blade of this species.

#### Distribution.

**Figure 7.**
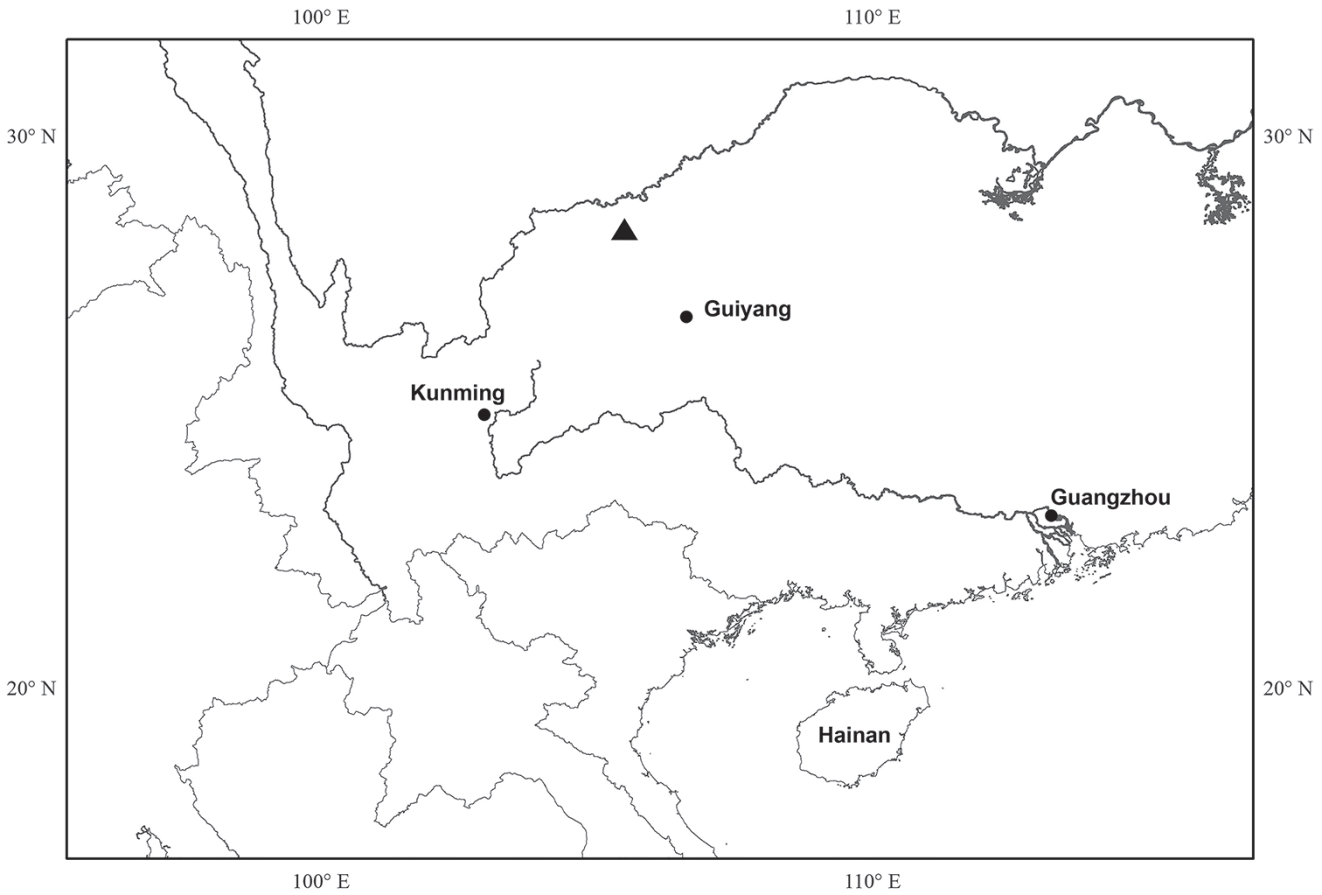
Distribution of *Bredia hispida* (triangle).

*Bredia hispida* is currently known from Xuyong County, southeastern Sichuan, China (Fig. 7). It occurs on damp steep red sandstone cliff, often below a seasonal waterfall, at 1000–1400 m.

#### Additional specimen examined.

China. Sichuan: Xuyong County, Shui-wei town, Guan-dou village, 15 Sept 2013, W. B. Ju and H. N. Deng, HGX13524 (CDBI); Xuyong County, Shui-wei town, Guang-mu village, 27 Aug 2013, W. B. Ju and H. N. Deng, HGX12702 (CDBI); Xuyong County, Long-feng town, Ling-guan-ti power station, 4 Aug 2012, X. F. Gao, Y. D. Gao and W. B. Ju, HGX10961 (CDBI).

## Supplementary Material

XML Treatment for Bredia hispida

## Supplementary materials

Supplementary material 1 Table S1. Source of materials studied and GenBank accession numbers for nrITS Data type Table Explanation note Newly generated sequences are indicated in bold. https://binary.pensoft.net/file/428587This dataset is made available under the Open Database License (http://opendatacommons.org/licenses/odbl/1.0/). The Open Database License (ODbL) is a license agreement intended to allow users to freely share, modify, and use this Dataset while maintaining this same freedom for others, provided that the original source and author(s) are credited.Jin-Hong Dai, Qiu-Jie Zhou, Ren-Chao Zhou, Ying Liu

Supplementary material 2 Table S2. Summary statistics of sequences used for phylogenetic analyses Data type Table Explanation note PIS, parsimony-informative sites. https://binary.pensoft.net/file/428588This dataset is made available under the Open Database License (http://opendatacommons.org/licenses/odbl/1.0/). The Open Database License (ODbL) is a license agreement intended to allow users to freely share, modify, and use this Dataset while maintaining this same freedom for others, provided that the original source and author(s) are credited.Jin-Hong Dai, Qiu-Jie Zhou, Ren-Chao Zhou, Ying Liu

Supplementary material 3 Table S3. Pairwise genetic distances among species of *Bredia* at the nrITS region Link: https://doi.org/10.3897/phytokeys.152.53512.suppl3 Data type Table Explanation note Those between B. hispida and remaining species are indicated in bold. https://binary.pensoft.net/file/428589This dataset is made available under the Open Database License (http://opendatacommons.org/licenses/odbl/1.0/). The Open Database License (ODbL) is a license agreement intended to allow users to freely share, modify, and use this Dataset while maintaining this same freedom for others, provided that the original source and author(s) are credited.Jin-Hong Dai, Qiu-Jie Zhou, Ren-Chao Zhou, Ying Liu
